# Proinflammatory synergy between protease and superantigen streptococcal pyogenic exotoxins

**DOI:** 10.1128/iai.00405-24

**Published:** 2025-01-29

**Authors:** Anders F. Johnson, Summer D. Bushman, Doris L. LaRock, Juan Manuel Díaz, John K. McCormick, Christopher N. LaRock

**Affiliations:** 1 Department of Microbiology and Immunology, Emory University School of Medicine 12239 , Atlanta, Georgia, USA; 2 Microbiology and Molecular Genetics Graduate Program, Emory University 1371 , Atlanta, Georgia, USA; 3 Division of Infectious Diseases, Department of Medicine, Emory University School of Medicine 12239 , Atlanta, Georgia, USA; 4 Department of Microbiology and Immunology, University of Western Ontario 6221 , London, Ontario, Canada; St Jude Children's Research Hospital, Memphis, Tennessee, USA

**Keywords:** *Streptococcus pyogenes*, group A *Streptococcus*, superantigens, toxin-mediated diseases, proteases

## Abstract

Streptococcal pyogenic exotoxins (Spe proteins) secreted by *Streptococcus pyogenes* (group A *Streptococcus*, GAS) are responsible for scarlet fever and streptococcal toxic shock syndrome. Most Spes are superantigens that cause excessive inflammation by activating large numbers of T cells. However, Streptococcal pyogenic exotoxin B (SpeB) is an exception, which is pro-inflammatory through its protease activity. Prior work shows that SpeB has the potential to cleave bacterial proteins. If cleavage of superantigens results in their inactivation, this gives the possibility that these two classes of exotoxins work at cross-purposes. We examined SpeB cleavage of the 11 major GAS superantigens and found that lability was not specific to structure, conservation, or, when compared to orthologous superantigens from *Staphylococcus aureus*, species of origin. We further show that rather than strictly antagonizing superantigen activity through degradation, SpeB can synergistically enhance superantigen-induced inflammation. For SpeB-labile superantigens, such as SmeZ, this is limited due to degradation, but for protease-resistant superantigens like SpeA, activity remains synergistic even at high protease concentrations. These findings suggest two modes by which proteases like SpeB may post-translationally regulate superantigens: positively, as a force amplifier that cooperatively increases inflammation, and negatively, through degradation that could act as a rheostat-like mechanism to limit excessive immune activation. Both mechanisms may contribute to the pathogenesis of GAS and other superantigen-producing pathogens.

**IMPORTANCE:**

*Streptococcus pyogenes* produces both superantigen and protease virulence factors to subvert host immunity. However, its major protease is highly promiscuous and would potentially limit superantigen activity through its degradation. We profile the sensitivity of the streptococcal superantigens to degradation by the protease SpeB, providing evidence that many are highly resistant. Furthermore, we show that these important toxins can have synergistic proinflammatory activity. This provides insight into diseases like scarlet fever and toxic shock syndrome caused by these toxins and suggests anti-inflammatories that may be therapeutically useful.

## INTRODUCTION

Group A *Streptococcus* (GAS) is an obligate human pathogen responsible for an estimated 600,000 deaths annually (1). Beyond pharyngitis, GAS is also responsible for serious infections such as scarlet fever (SF), necrotizing fasciitis, and streptococcal toxic shock syndrome (STSS). Severe GAS infections require rapid and intensive treatment (2); in addition to antibiotics (3), intravenous immunoglobulin has shown protective effects (4), and surgical intervention by removal of infected tissue has been a mainstay for necrotic skin and soft tissue infections (5). There is no approved vaccine for GAS (6), but a greater understanding of disease pathogenesis can give targets for therapeutic strategies and vaccine development.

SF and STSS are specifically caused by secreted streptococcal pyogenic exotoxin (Spe) proteins that act as superantigens (1). Superantigens are T cell mitogens that bypass the antigen-processing pathway by directly binding both T cell receptors and the MHC-II receptors of antigen-presenting cells (7). This results in cell activation unconstrained by antigen specificity, leading to a large proportion of cells becoming activated and causing a cytokine storm through the release of proinflammatory cytokines such as IL-1β, IL-6, and IFN-γ (8, 9). While this is a major driver of severe disease, the specific mechanisms by which GAS derives fitness benefits under typical circumstances are not yet entirely clear. Superantigens are thought to promote colonization and dissemination (10–12) and may remodel host immunity to be more permissive to recurrent or persistent infections by promoting T cell anergy and killing of B cells (13, 14).

Streptococcal pyogenic exotoxin B (SpeB) is another conserved and essential virulence factor of GAS. Unlike other Spe proteins, SpeB is a cysteine protease (15) and not a superantigen (16). SpeB is one of the most abundant secreted proteins of GAS during culture and infection (17–19) and acts to broadly inactivate immune antimicrobials and promote tissue dissemination (20). SpeB also strongly drives pathology and inflammation (21, 22), in part by directly activating inert IL-1β and IL-18 into their active proinflammatory forms (23, 24). SpeB and superantigens were originally isolated as the causative agents behind scarlet fever (19), but the extent to which these proteins promote each other’s activities remains unclear. SpeB has been shown to be capable of cleaving most of the GAS proteome (17), giving the possibility that it broadly antagonizes superantigen function. In counterpoint to this, both SpeB and superantigens are required across similar infection models and are broadly conserved (10, 25–29), suggesting their activities are not mutually exclusive. In a more nuanced view, this may be variable between superantigens, as the highly conserved superantigen SmeZ is readily degraded by SpeB (30), while SpeA, a phage-encoded superantigen associated with severe disease, is highly resistant (31). This suggests that each superantigen may have different sensitivity to SpeB cleavage, but the resistance profile of the remaining streptococcal superantigens is unknown.

Here, we examine the proinflammatory signaling contributing to superantigen reactivity and show that cytokines activated by SpeB enhance the superantigen response. Additionally, we examine the resistance of each of the GAS superantigens to degradation by the protease SpeB. Altogether, these results support a model wherein lower concentrations of SpeB and superantigens can synergize for enhanced efficacy. Conversely, degradation of labile superantigens upon high expression of SpeB may serve as a brake to limit excessive toxicity. Furthermore, the inhibition of inflammatory pathways activated by SpeB can help limit cellular responses to superantigens.

## RESULTS

### SpeB-activated cytokines promote the activation of T cells by superantigens

**Fig 1:**
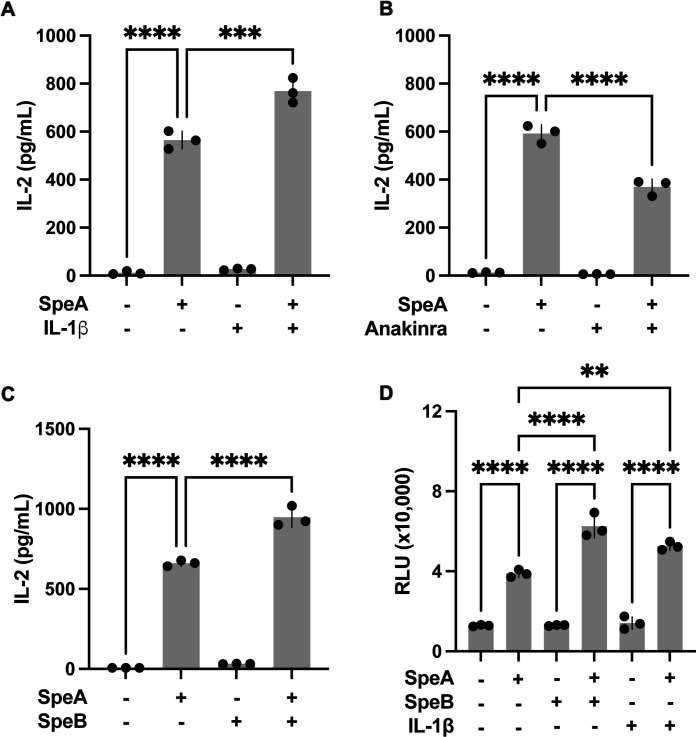
SpeB synergizes with superantigens to enhance activity. Human PBMCs were incubated for 24 hours with recombinant SpeA (10 ng), human IL-1β (1 ng) (**A**), SpeA (10 ng) and IL-1 inhibitor Anakinra (1 μg) (**B**), SpeB (100 ng) and SpeA (10 ng) (**C**) and then the supernatant IL-2 was measured by ELISA. After 6 days of incubation under these conditions, cell proliferation and viability were evaluated by CellTiter-Glo 2.0 assay (**D**). Data values are from three independent experiments. Bars show median values ± standard error of the mean. Statistics were examined by repeated measures ANOVA with Tukey post-test, ***P < 0.0005 and ****P < 0.00005.

The cytokine IL-1β is strongly activated during GAS infections, which has established epidemiological and mechanistic links to pathological inflammation (23, 25, 32, 33). This cytokine has broad immune activation roles, including the priming of antigen-presenting cells and T cells (34–36), which have the potential to alter their sensitivity to superantigens. We used the established human peripheral blood mononuclear cell (PBMC) model where IL-2 is a sensitive and specific marker of superantigen-activated T cells (9, 16, 37–39). SpeA significantly induced IL-2 production from human PBMCs (Fig. 1A), as in prior works. This was significantly increased with the addition of exogenous IL-1β at concentrations observed during infection (23, 25), which had no impact in the absence of SpeA (Fig. 1A). Additionally, the inclusion of Anakinra, a drug that acts as a competitive antagonist that fully blocks IL-1 signaling when in excess, decreased IL-2 production in response to SpeA (Fig. 1B). Since SpeB is strongly pro-inflammatory through its maturation of IL-1β and processing of other substrates (20), we next examined the impact of SpeB on IL-2 production. Alone, SpeB did not induce IL-2, but it heightened the response to SpeA (Fig. 1C). Similarly, cell proliferation induced by SpeA was enhanced by the addition of SpeB or IL-1β (Fig. 1D). Together, these data suggest a role for SpeB-induced IL-1β in the amplification of the T cell response to the superantigen SpeA.

### SpeB has a synergistic effect on superantigen activity

**Fig 2:**
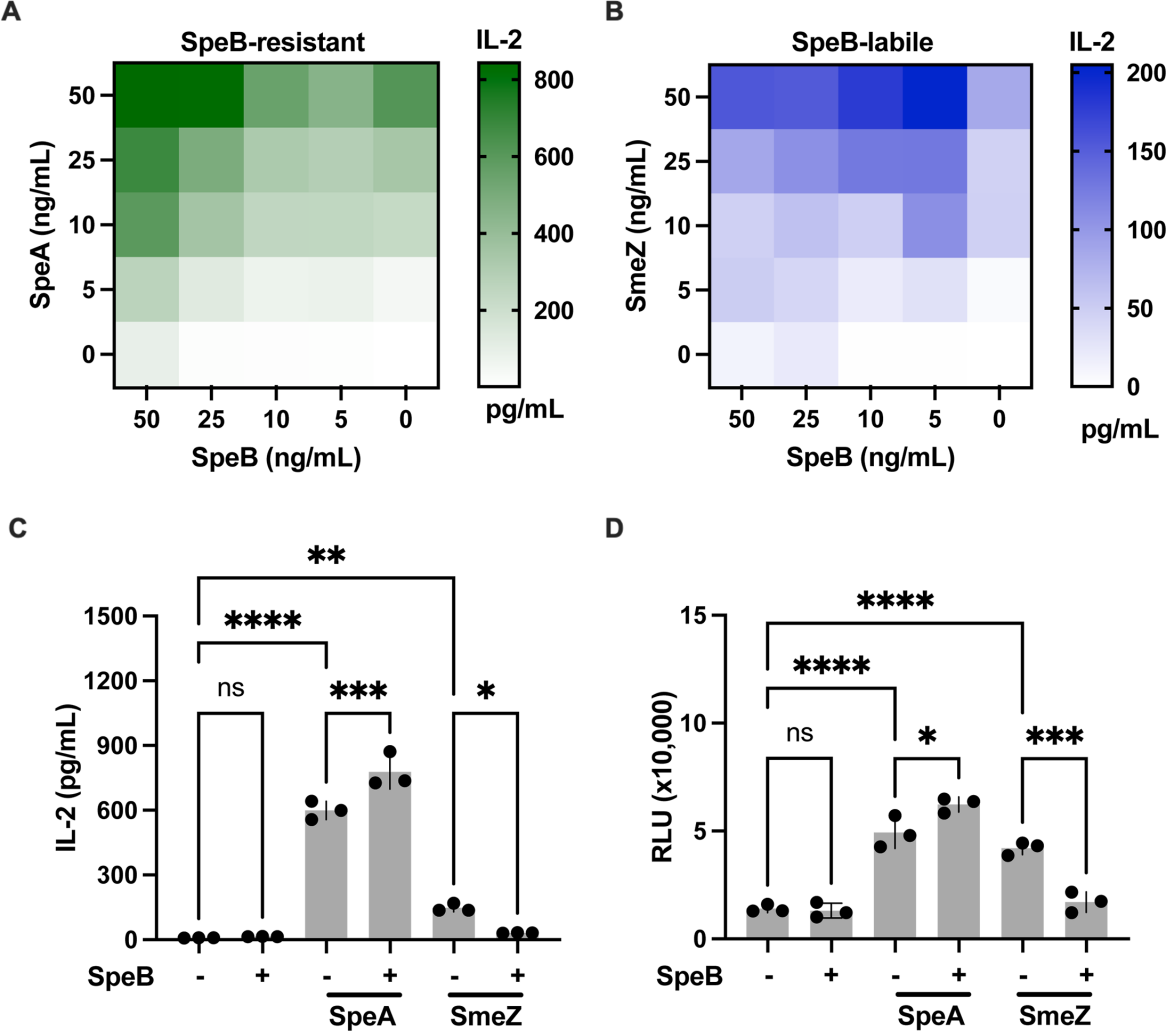
SpeB synergizes with superantigens to enhance activity. Superantigens and SpeB were incubated with PBMCs at the indicated concentration and combination for 24 hours. Heatmap shows the concentration of IL-2 in the supernatant, quantified by ELISA, in response to SpeA (**A**) and SmeZ (**B**). Human PBMCs were incubated with recombinant SpeB (100 ng) and SpeA (10 ng) or SmeZ (10 ng) and then IL-2 was quantified after 1 day (**C**) or cell proliferation after 6 days (**D**). Data are pooled from three independent experiments. Bars show median values ± standard error of the mean. Statistics were examined by repeated measures ANOVA with Tukey post-test, ***P < 0.0005 and ****P < 0.00005.

We next sought to examine whether degradation by SpeB is a limitation to superantigen responses, taking advantage of SmeZ, which unlike SpeA (31), is readily degraded (30). Using the human PBMC model, we concurrently incubated cells with SpeB and either SpeA or SmeZ in a checkerboard format of varying concentrations. Superantigen activity was quantified by ELISA using IL-2 as a measurement of T-cell activation. In the absence of SpeB, there were dose-dependent IL-2 responses to SpeA (Fig. 2A) and SmeZ (Fig. 2B). In the absence of superantigens, SpeB did not induce significant amounts of IL-2. For every concentration of SpeA, increasing quantities of SpeB increased IL-2 production by 137%–570%, with the largest additive effect at the lowest superantigen concentrations. SpeB-dependent increases in IL-2 production of 228%–550% were also seen with SmeZ. However, this only occurred with the lowest concentration (5 ng/mL) of SpeB. Beyond this point, increasing SpeB only leads to dose-dependent decreases in activity. When SmeZ is predigested by incubation with SpeB for 24 hours before addition to PBMCs, the ability to induce IL-2 (Fig. 2C) or cell proliferation (Fig. 2D) is fully lost, while SpeA’s is maintained. Taken together, these data show that SpeB has the potential to act synergistically with streptococcal superantigens like SpeA, but that this synergy can be limited if the superantigen is degraded by SpeB like SmeZ.

### Degradation of streptococcal superantigens by SpeB is not associated with recent superantigen gene acquisition

**Fig 3:**
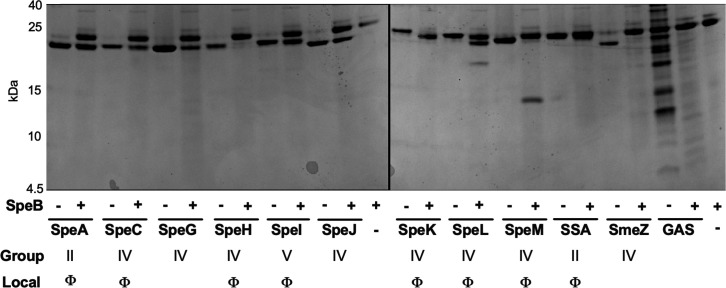
Variable susceptibility of GAS superantigens to SpeB degradation. One microgram of each recombinant GAS superantigen (SpeA, SpeC, SpeG, SpeH, SpeI, SpeJ, SpeK, SpeL, SpeM, SSA, and SmeZ) was incubated with SpeB (1 μg) or SpeB^−^ negative control for 24 hours at 37°C. A boiled crude protein extract was included as a control for SpeB activity. Proteins and cleavage products were separated by SDS-PAGE and visualized by staining. Each superantigen is labeled for the evolutionary group to which it belongs and whether it is prophage associated (Φ). Data are representative of three independent repeats.

While SpeB proteolytic activity toward SpeA and SmeZ has been characterized (30, 31), activity toward the other streptococcal superantigens remains largely unknown. To better understand the limitations to which there is cooperation between Spe proteins, we sought to identify whether SpeB broadly degrades superantigens, like it does SmeZ, or if they are more typically resistant, like SpeA. Thus, using a panel of purified GAS superantigens, we incubated them individually with SpeB and used SDS-PAGE to visualize any cleavage products formed. SpeB proteolytic activity was confirmed by hydrolysis of the specific substrate IFFDTWDNE as previously (23) and degradation of a loading control of a GAS protein extract (Fig. 3). Consistent with prior results (30, 31) and our earlier experiments, SpeA was not cleaved by SpeB under conditions where there was complete degradation of SmeZ (Fig. 3). Targeting by SpeB was highly variable between the 11 superantigens: SpeH, SpeK, and SmeZ were fully degraded, SpeC and SpeG were partially degraded, SpeL and SpeM had distinct cleavage products that were not further degraded, and no change was observed for SpeA, SpeI, SpeJ, or streptococcal superantigen (Fig. 3). Altogether, there was no distinct commonality as to whether a superantigen was susceptible to SpeB based on whether it is known to be conserved in the core genome or a variable between strains and possibly more recently horizontally acquired (40).

### Degradation by SpeB is variable among group IV superantigens

**Fig 4:**
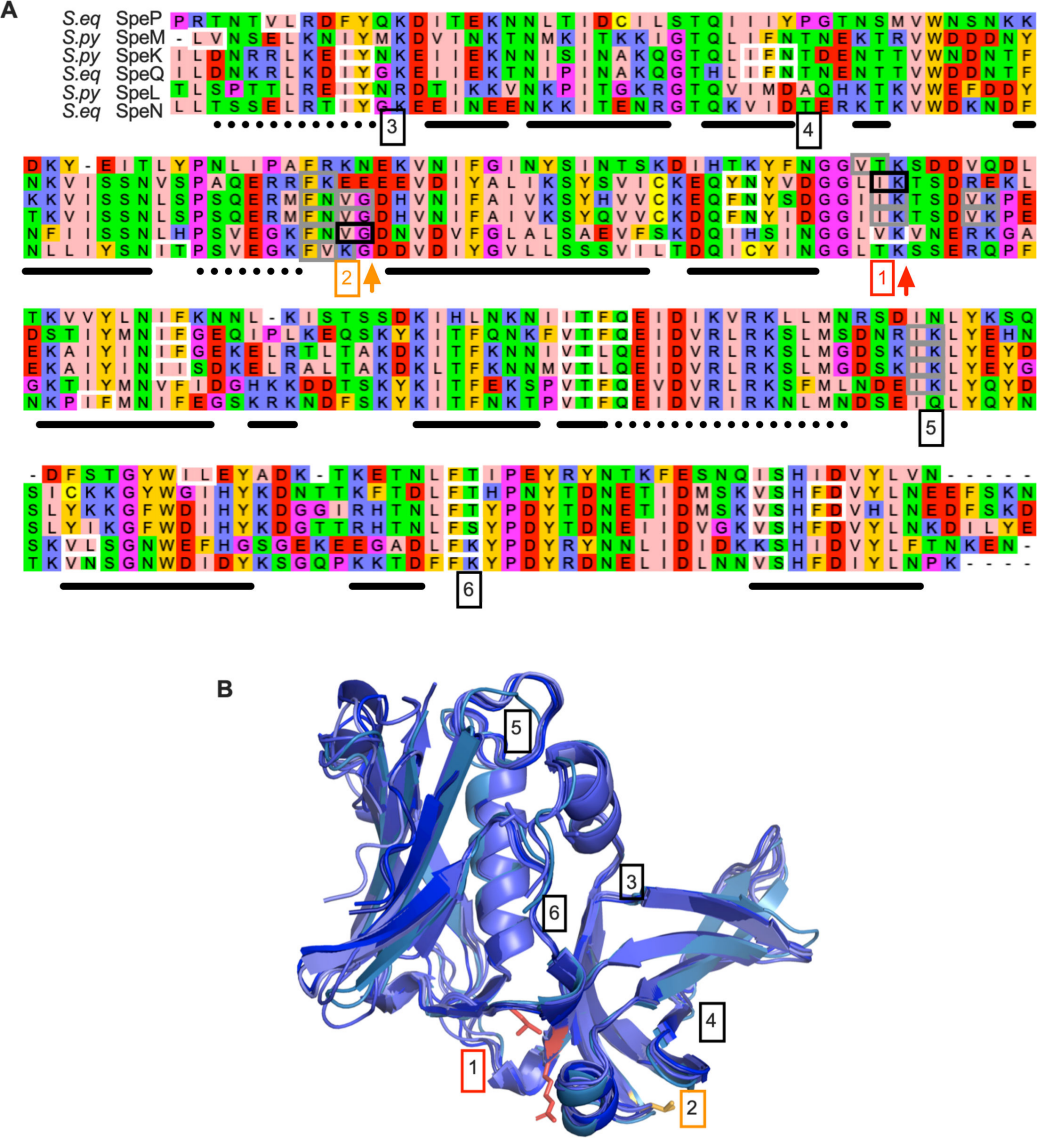
SpeB cleavage of class IV superantigens. (**A**) SpeM and SpeL aligned to related superantigens, with Edman degradation-verified SpeB cleavage sites boxed in black and highlighted in [1], red, and [2], orange. Boxed in gray are conserved cleavage sites in proteins not tested. Potential sites where no cleavage was observed are boxed in white in the sequence alignment and highlighted as [3–6] in the aligned structure (**B**).

Most GAS superantigens show sequence homology and structural identity relatedness that categorizes them as members of superantigen Group IV (41). SmeZ and SpeH, the most divergent from this group, are both degraded by SpeB, as is SpeK; SpeC, SpeG, and SpeJ are more SpeB resistant, and SpeL and SpeM share a pattern of limited proteolysis (Fig. 3). To better understand which sites in these related proteins are recognized by SpeB, the cleavage products were sequenced by Edman degradation, identifying cleavage of SpeL after Val-Gly-119 and SpeM at Ile-Lys-122 (Fig. 4). These match expected cleavage sites based on the substrate amino acid specificity profile of SpeB (42–44). However, there were a number of predicted cleavage sites that did not yield a fragment during incubation with SpeB (Fig. 4A). These are likely located in regions inaccessible due to protein folding (Fig. 4B) since the catalytic pocket of SpeB can only accommodate extended loop or disordered regions within its natural substrates (23, 24, 26, 43, 45). The site cleaved in SpeL, Val-Gly-119, is Glu-Glu in SpeM. This is not a viable cleavage site based on biochemical studies of SpeB (42–44), matching our observation that SpeB does not cleave SpeM at that site. Additionally, the SpeM cleavage site Ile-Lys-122 is Val-Lys in SpeL; this Ile-to-Val substitution is predicted to decrease SpeB cleavage by at least fourfold in idealized substrates (44). Since for each cleavage site, there is a superantigen that is active despite lacking that site, there may not be selection against the SpeB cleavage sites within SpeL and SpeM. Consistent with this, a similar distribution of these identified cleavage sites is present in the highly similar *Streptococcus equi* superantigens SpeN, SpeO, and SpeQ, despite *S. equi* not encoding a SpeB homolog (Fig. 4A).

### Degradation of class II staphylococcal and streptococcal superantigens

**Fig 5:**
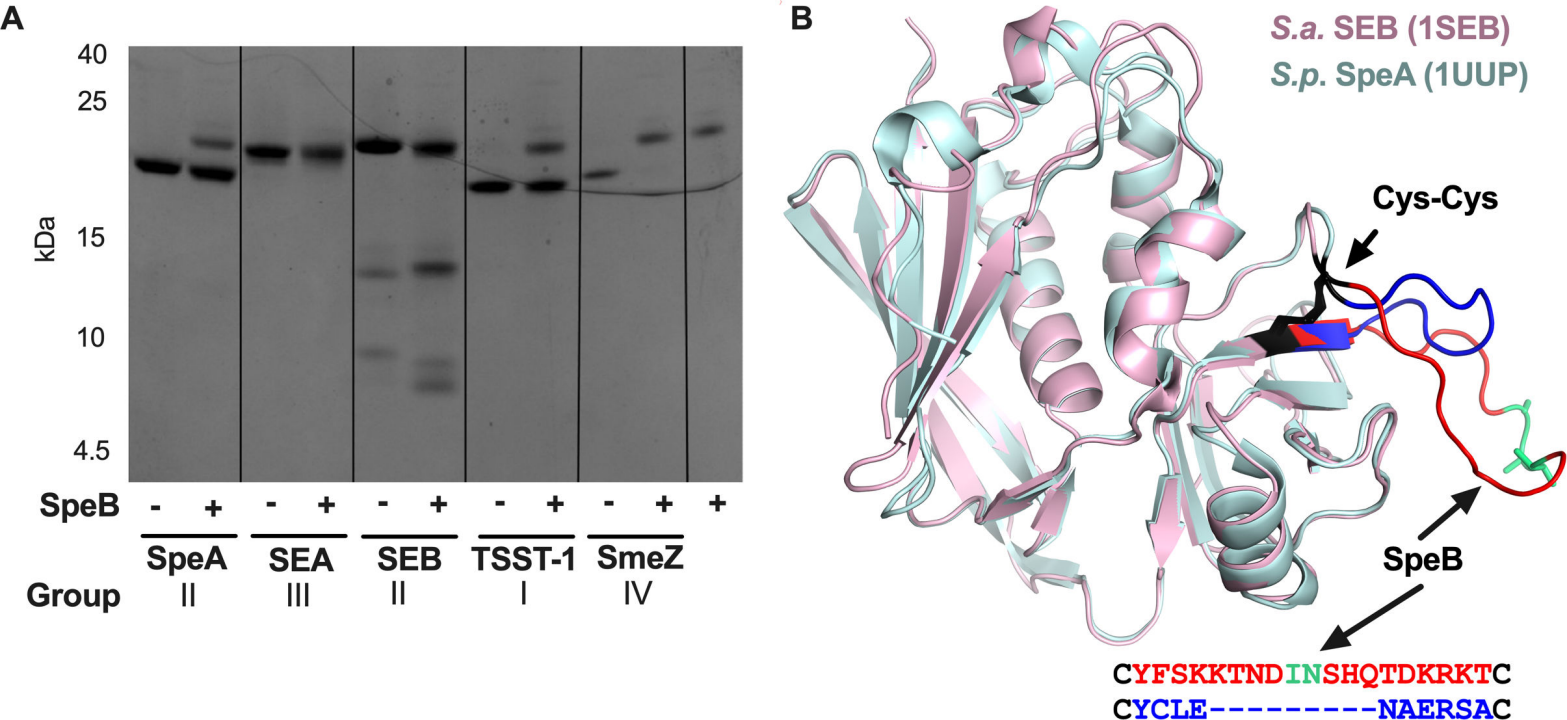
SpeB degradation of staphylococcal superantigens is variable. (**A**) One microgram of SpeA, SEA, SEB, TSST-1, or SmeZ was incubated with 1 μg of SpeB for 24 hours at 37°C, with SpeB^−^ negative control, then cleavage products were visualized by SDS-PAGE and staining. Data are representative of three independent repeats. (**B**) SEB (1SEB, pink) and SpeA (1UUP, light blue) aligned in Pymol, with the SpeB cleavage site in SEB (after Asn131) confirmed by Edman sequencing highlighted in teal.

To better understand the relationship between SpeB cleavage of a superantigen and species of origin, we next examined the major characterized superantigens of *S. aureus*. Because they did not evolve in the presence of SpeB, they may not have had selective pressure to minimize accessible SpeB cleavage sites and may be more susceptible to SpeB degradation. Staphylococcal exotoxin A (SEA) and Toxic shock syndrome toxin-1 (TSST-1) are superantigens belonging to classes III and class I, which are absent from GAS; SpeB degraded SEA but had no activity toward TSST-1 (Fig. 5A). Of interest, we observed cleavage of SEB but not SpeA (Fig. 5A). These group II superantigens share significant sequence and structural homology (46), and each is historically linked to severe disease like toxic shock syndrome in their respective species (8). Edman degradation was used to identify the SpeB cleavage site within SEB, which was confirmed to be after Ile-Asn131 (Fig. 5B), consistent with other identified SpeB cleavage sites (23, 24, 26, 43, 45). The location of this cleavage site is notable for occurring within an extended cysteine loop absent in SpeA that is thought to be responsible for the emetic activity of SEB during food poisoning (47, 48). SpeA is not emetic (49) and has the equivalent cysteine loop truncated by nine amino acids relative to SEB (Fig. 5B), thus removing the site that allows degradation by SpeB.

## DISCUSSION

Expression of superantigens and SpeB is highest in culture as the bacteria are entering the stationary phase (50). In human infections, as well as non-human primate and mouse infection models, superantigen and SpeB expression track and are among the most highly induced secreted virulence factors (51–54). Interactions will occur due to this temporal and spatial co-occurrence, and, given their individual requirements for infection, will have consequences that may differ by specific superantigen, bacterial strain, anatomical location, and disease stage (55). Thus, identifying which superantigens are susceptible to degradation by SpeB is the first step to understanding this potentially dynamic process.

There are five distinct superantigen groups based on function and structural makeup, each group containing several related members (56). While, in part, this variability may limit the generation of neutralizing antibodies, each superantigen binds variable TCR and MHC class II molecules with different affinities, potentially maximizing activation when accounting for the diversity in the human population. We show that these superantigens are also variable in their lability to the ubiquitous, conserved, and essential protease SpeB (Table S1). Three superantigens, SmeZ, SpeG, and SpeJ, are most conserved among GAS, while the pan-genome contains at least eight others, carried on mobile elements, and present in variable combinations in each strain (57). Since the core-genome superantigen SmeZ was known to be degraded while the prophage-encoded and virulence-associated superantigen SpeA was known to be resistant, we anticipated that SpeB resistance might contribute to the selection for new superantigen acquisition. However, 63% (5/8) of the strain-variable superantigens had significant resistance to SpeB, similar to the 66% (2/3) resistance observed in the conserved superantigens.

We also considered the possibility that there was selection to maintain SpeB cleavage sites with superantigens. Since superantigen activity can be lost with higher quantities of SpeB, post-transcriptional regulation of superantigen function through proteolysis by SpeB provides a plausible mechanism for GAS to modulate their levels and prevent overstimulation of the host immune system. An abundance of non-degradable superantigens would then be expected to be linked to more severe inflammation and disease. Such a mechanism would provide a basis for the associations of SpeA, SSA, and SpeC in outbreaks of scarlet fever (38); SpeA and SSA are SpeB resistant, and SpeC is degraded at a slow rate. Strains with increased production of one or more of these SpeB-resistant superantigens would also be expected to be more proinflammatory. This is seen in the M1_UK_ lineage, which expresses greater SpeA and is associated with the superantigen-mediated disease scarlet fever (58). The corollary to these points is that strains deficient in SpeB would not gain a synergy boost and would lose post-translational regulation by degradation. Superantigen inactivation is most likely to occur at cell densities with the greatest risk of superantigen overproduction, SpeB is induced by quorum sensing at high cell densities of Streptococci (59). Mutations that block SpeB expression naturally arise during natural human and animal models of invasive infections (20). Under these circumstances, it would be expected that SpeB-sensitive superantigens that would otherwise be cleaved (SpeH, SpeK, SmeZ, SpeL, and SpeM) would accumulate and contribute to STSS and other inflammatory complications. These observations may carry to other pathogens such as *S. aureus*; it also produces several highly active secreted proteases, including ScpA, SspA, SspB, and aureolysin (60), with the potential to regulate or synergize with the superantigens it produces.

Furthermore, we demonstrate that SpeB can enhance the activity of superantigens and that this increase is beyond additive. Even with SmeZ, a superantigen established as very susceptible to degradation by SpeB (30), a synergistic effect is observed. Prior work has shown that SpeB can increase GAS pathogenesis through IL-1 family cytokines (25) and the induction of other, uncharacterized, inflammatory processes (22). It is expected that cytokines activated by SpeB act in tandem with the cytokines induced by superantigens, as part of GAS’s broadly proinflammatory virulence strategy (10–12). However, since the SpeB-activated cytokines IL-1β and IL-18 also contribute to the poise and activation of superantigen-responsive T cell subsets (61–64), the convergence of pathways may be a synergistic process for maximal inflammation from minimal toxin production.

GAS remains broadly antibiotic susceptible; invasive infections are often non-responsive to antimicrobial monotherapy and require additional treatment, including surgical removal of infected tissue (3). The morbidity and mortality observed are not just damage caused by the microbe but from toxin-mediated inflammation. Understanding the genesis of the cytokine storm observed in severe disease allows for selective inhibition of the most pathological processes with immunomodulatory drugs (20, 65). Host-directed therapeutics may be useful for treating diseases, and several existing ones inhibit these SpeB- and superantigen-induced inflammatory pathways (65–68). In tandem with antibiotics and other interventions, such as surgery and intravenous immunoglobulin, immunotherapies to control inflammation may help reduce pathology, buy additional time during sepsis and shock, and improve patient outcomes.

## MATERIALS AND METHODS

### Protein purification

SpeB was purified as previously described (69), with >99% purity shown by SDS-PAGE and quantified by Bradford assay. Recombinant streptococcal superantigen SpeA2 was cloned from GAS M1T1 5448. The coding sequence, minus the signal peptide, for SpeA2 was generated for expression in pET-SUMO. Expression was induced from BL21 *Escherichia coli* with 1 mM IPTG (Research Product International) for 3 hours at 37°C. Cells were pelleted at 8,000 *g* for 10 minutes and then frozen at −20°C until use. Cell pellets were thawed and resuspended in PBS, lysed by sonication at 15% amplitude (Sonic Dismembrator Model 500, Fisher Scientific) for 4 minutes at 30 second intervals on ice, and then centrifuged at 21,000 *g* for 30 minutes at 4°C. Lysate was run through Talon gravity columns with Cobalt resin (Thermo Scientific), washed with PBS, and eluted in PBS with 300 mM imidazole (Sigma). Protein was dialyzed overnight at 4°C in PBS with ulp1 protease to remove the SUMO tag and imidazole and then purified by reverse nickel column. The ulp1 protease was generated previously (26). Recombinant streptococcal superantigens SpeC, SpeG, SpeH, SpeI, SpeJ, SpeK, SpeL, SpeM, SmeZ, and SSA were generated previously (10). Recombinant staphylococcal superantigens SEA, SEB, and TSST-1 were purchased lyophilized (Toxin Technologies, FL, USA) and resuspended in PBS.

### Primary cell culture

Peripheral blood mononuclear cells were isolated from healthy human donor blood by centrifugation over Ficol Histopaque 1077 (Sigma) and frozen at 5 × 10^6^ cells/mL in 90% FBS/10% DMSO until use. Cells were thawed and cultured in RPMI + 10% heat-inactivated FBS (Atlanta Biologicals). Cells were maintained at 37°C and 5% CO_2_. PBMCs were cultured in 100 µL volumes at 1 × 10^5^ cells/well in tissue culture-treated 96-well plates. Superantigens and SpeB were added from 100× stocks to the indicated concentrations and then incubated at 37°C and 5% CO_2_. After 24 hours, supernatants were then collected, quantified, and analyzed by ELISA for IL-2 following the manufacturer’s protocol and recommendations for dilutions (R&D Biosciences). IL-1 activity was measured by bioassay as previously (66). Evaluation of cell proliferation requires a longer duration (70), so incubations were extended to 6 days and followed by cell quantification by CellTiter-Glo 2.0 assay following the manufacturer’s protocol (Promega).

### SDS-PAGE

Superantigens, 1 µg each, were incubated with 1 µg SpeB in PBS containing 2 mM Dithiothreitol (Sigma) at 37°C. Reactions were stopped by freezing at −20°C. Samples were prepared for SDS-PAGE with tricine SDS sample buffer (Invitrogen) and sample reducing agent (Invitrogen) and heated to 85°C for 2 minutes. Samples were separated on 16% Tricine gels (Invitrogen), protein stained with AquaStain (Bulldog Bio), and visualized. Then, the intensity of bands in SpeB-treated samples relative to the untreated control was examined by densitometry in Image Lab (Bio-Rad) to examine processivity and differentiate between proteins fully cleaved or fully resistant. New amino termini generated by SpeB cleavage were identified by Edman degradation as previously (23). Briefly, SDS-PAGE separated proteins were wet-transferred to the PVDF membrane, visualized with SimplyBlue Safe Stain (Invitrogen), and then sequenced on an ABI 494 protein sequencer by the Tufts University Core Facility.

### Protein modeling and predictions

Prior structures of SEB (71) and SpeA (72) and predicted structures of all other superantigens from the Alphafold Structure Database (73) were aligned and visualized in PyMOL version 2.5.2 (Schrodinger) from the KC906583, AAL97848, KC906584, NP607350, NP795602, and DAC73996.1 reference sequences defined previously (41). Amino acid sequences were aligned in Lasergene version 17 (DNAstar). Previously defined substrate cleavage sites of SpeB (23, 24, 26, 42, 44, 45) were aligned to define the motif [IVFYM]-[ETAGSND] with the included condition for a net negative charge sidechain charge in the P1′–P5′ region (the five residues C-terminal to the cleavage site), then queried using ScanProsite (Expasy).

### Statistics and data analysis

Values shown are expressed as mean ± standard deviation. Differences between groups were analyzed by repeated measures ANOVA with Tukey post-test. Significance was assigned to *P* values of <0.05, calculated with Prism version 10 (GraphPad).

## SUPPLEMENTAL MATERIAL

Table S1 iai.00405-24-s0001.docx Summary of superantigen susceptibility to SpeB.

ASM does not own the copyrights to Supplemental Material that may be linked to, or accessed through, an article. The authors have granted ASM a non-exclusive, world-wide license to publish the Supplemental Material files. Please contact the corresponding author directly for reuse.
