# Supplementary material for: Proinflammatory synergy between protease and superantigen streptococcal pyogenic exotoxins
Source: Infect Immun. 2025 Jan 29;93(3):e00405-24. doi: 10.1128/iai.00405-24 (PMC11895496; doi:10.1128/iai.00405-24)
Supplement: Table S1 — Summary of superantigen susceptibility to SpeB. [file iai.00405-24-s0001.docx]

**Table1.** Summary of Superantigen Susceptibility to SpeB

| Superantigen | Species | Class | SpeB-processing |
| --- | --- | --- | --- |
| SEA | *S. aureus* | III | Degraded |
| SEB | *S. aureus* | II | Degraded |
| SmeZ | *S. pyogenes* | IV | Degraded |
| SpeA | *S. pyogenes* | II | Resistant |
| SpeC | *S. pyogenes* | IV | Limited degradation |
| SpeG | *S. pyogenes* | IV | Limited degradation |
| SpeH | *S. pyogenes* | IV | Degraded |
| SpeI | *S. pyogenes* | V | Resistant |
| SpeJ | *S. pyogenes* | IV | Resistant |
| SpeK | *S. pyogenes* | IV | Degraded |
| SpeL | *S. pyogenes* | IV | Limited cleavage |
| SpeM | *S. pyogenes* | IV | Limited cleavage |
| SSA | *S. pyogenes* | II | Resistant |
| TSST-1 | *S. aureus* | I | Resistant |
